# Nectin-4 Expression Is an Independent Prognostic Biomarker and Associated With Better Survival in Triple-Negative Breast Cancer

**DOI:** 10.3389/fmed.2019.00200

**Published:** 2019-09-13

**Authors:** Jasmin Zeindler, Savas Deniz Soysal, Salvatore Piscuoglio, Charlotte K. Y. Ng, Robert Mechera, Andrej Isaak, Walter Paul Weber, Simone Muenst, Christian Kurzeder

**Affiliations:** ^1^Breast Center, University Hospital Basel and University of Basel, Basel, Switzerland; ^2^Visceral Surgery Research Laboratory, Clarunis, Department of Biomedicine, University of Basel, Basel, Switzerland; ^3^Department of Surgery, Clarunis University Center for Gastrointestinal and Liver Diseases Basel, Basel, Switzerland; ^4^Institute of Medical Genetics and Pathology, University Hospital Basel, Basel, Switzerland; ^5^Department of Vascular and Endovascular Surgery, University Hospital Basel, Basel, Switzerland; ^6^Department of Obstetrics and Gynecology, University Hospital of Basel, Basel, Switzerland

**Keywords:** Nectin-4, breast cancer, biomarkers, prognosis, triple-negative breast cancer

## Abstract

**Background:** Triple-negative breast cancer (TNBC) represents about 10-20% of all invasive breast cancers and is associated with a poor prognosis. The nectin cell adhesion protein 4 (Nectin-4) is a junction protein involved in the formation and maintenance of cell junctions. Nectin-4 has previously shown to be expressed in about 60% of TNBC as well as in TNBC metastases, but to be absent in normal breast tissue, which makes it a potential specific target for TNBC therapy. Previous studies have shown an association of Nectin-4 protein expression with worse prognosis in TNBC in a small patient cohort. The aim of our study was to explore the role of Nectin-4 in TNBC and confirm its impact on survival in a larger TNBC patient cohort.

**Material and Methods:** We performed immunohistochemical staining for Nectin-4 on a tissue microarray encompassing 148 TNBC cases with detailed clinical annotation and outcomes data.

**Results:** A high expression of Nectin-4 was present in 86 (58%) of the 148 TNBC cases. In multivariate survival analysis, high expression of Nectin-4 was associated with a significantly better overall survival when compared with low expression of Nectin-4 (*p* < 0.001). Nectin-4-high expression was also significantly associated with a lower tumor stage (*p* = 0.025) and pN0 lymph node stage (*p* = 0.034).

**Conclusion:** Our results confirm that expression of Nectin-4 serves as a potential prognostic marker in TNBC and is associated with a significantly better overall survival. In addition, Nectin-4 represents a potential target in TNBC, and its role in molecular defined breast cancer subtype should be investigated in larger patient cohorts.

## Introduction

Triple-negative breast cancer (TNBC) is distinguished from other types of breast cancer by a particularly aggressive progression and poor clinical outcomes (1). Responsible for 10-20% of all invasive breast cancers, TNBC tends to affect younger women (2) and shows higher recurrence rates (1) as well as lower survival rates than non-TNBC (3). In fact, the 5-year survival rate for metastatic TNBC is <30%, while the overall survival rate declines close to zero (4). The poor prognosis associated with TNBC is largely owed to TNBC's “adverse” molecular characteristics, which considerably limit the scope of appropriate treatment options. With no expression of either estrogen or progesterone receptors, and no HER2 overexpression, TNBC cells lack the leverage points for efficient hormone therapy and/or HER2-targeted agents, which are successfully used for the treatment of non-TNBC. As a consequence, the only available treatment option for patients with TNBC is cytotoxic chemotherapy, often supplemented by the use of a platinum-based agent, which recent studies suggest enhances response to chemotherapy, especially in neo-adjuvant treatment settings (5). Pathologic complete response after neoadjuvant chemotherapy is considered one of the most important prognostic factors in early stage disease but can only been achieved in approximately one third of patients (6). Alternative treatment strategies in terms of molecularly targeted agents are thus desperately needed.

Although the rise of so-called “omics” technologies (such as genomics, transcriptomics, proteomics and metabolomics) over the past decade has significantly contributed to a better understanding of TNBC's molecular make-up, the search for actionable targets continues to be hampered by the striking molecular heterogeneity that TNBC displays (7). Gene expression analyses have shown that TNBC does not constitute a uniform disease entity to begin with, but can be classified into several subtypes with distinctive molecular ontologies (8). While the exact number of TNBC subtypes remains a subject of discussion, and can be expected to change as research progresses, the existence of at least four major TNBC subtypes seems by now fairly well-established, including a basal-like (BL), a mesenchymal (M), a luminal androgen receptor (LAR), and an immunomodulatory (IM) subtype. The most frequent TNBC subtype by far the BL subtype, accounts for about 70% of all TNBC cases (5, 9). TNBC's strong molecular heterogeneity would seem to offer sufficient potential leverage points for the design of molecularly targeted therapies; yet, despite the identification of several tumor-specific molecular alterations in various subtypes of TNBC, none of them has so far proven to be an actionable oncogenic driver for TNBC (8). At the same time, encouraging results have been achieved with the use of immunotherapeutic agents, e.g., monoclonal antibodies and cytokines, with increasing evidence to suggest the effectiveness of immunotherapy in at least some subgroups of TNBC patients (10, 11). A recent study demonstrated that enrichment levels of 26 immune cell activities were significantly higher in TNBC than in non-TNBC (12, 13). These findings, indicating an overall higher level of immunogenicity in TNBC compared to non-TNBC, underline the need for increased research on TNBC-specific cell surface molecules for the purpose of identifying new biomarkers as well as potential targets for immunotherapeutic agents.

One such TNBC-specific cell surface molecule is Nectin-4 (also known as PVRL4), which has previously been described as a new tumor-associated antigen in a number of different carcinomas (14–19). Being one of at least five members of the Nectin family, a group of cell adhesion molecules within the immunoglobulin superfamily, Nectin-4 consists of three conserved immunoglobulin-like domains (V, C, C) in its extracellular region. Unlike other Nectins, Nectin-4 is not expressed in normal adult tissue; however, several studies have found re-expression of Nectin-4 as a tumor-associated antigen in various cancer tissues, including pancreatic, ovarian, lung and breast cancer (14–19).

In this study, we further explore the role of Nectin-4 in a larger TNBC patient cohort.

## Methods

### Patients

We retrospectively recorded the clinicopathological features of all 148 patients included in this study, non-matched and non-stratified. The recorded features included patient age and gender, tumor localization, pT and pN stage, histological subtype, molecular subtype, BRE grade and overall survival. The median event-free follow-up time was 50.4 months. The treatments the patients received were according to the current guidelines at the time of treatment.

### Specimen Characteristics and Tissue Microarray Construction

We designed a tissue microarray (TMA) of 148 non-consecutive, primary human triple-negative breast cancers, sampled between 1985 until 2015. These samples were collected from the tissue biobank of the Institute of Pathology, University Hospital Basel, with the approval of the Ethics Committee Nordwestschweiz (EKNZ, Nr. 2014-396) and in compliance with ethical standards and medical confidentiality. Histological slides from all patients were collected from the archives of the Institute of Pathology, and all cases were reviewed by an experienced breast pathologist (S.M.). Diagnosis of TNBC was confirmed, and staining of ER, PR and Her2 was repeated when necessary, as described before, and in accordance with the ASCO/CAP guidelines (20). For each block, a morphologically representative area was identified, from which a tissue cylinder of 0.6 mm diameter was punched. Subsequently, a semi-automated tissue arrayer was used to assemble all cylinders into one recipient paraffin block, 30 × 25 mm in size. The punches were taken from the tumor center, thus ensuring that each TMA cylinder contained at least 50% of tumor tissue. The TMA blocks were stored in the certified biobank of the Institute of Pathology.

### Immunohistochemistry

**Figure 1:**
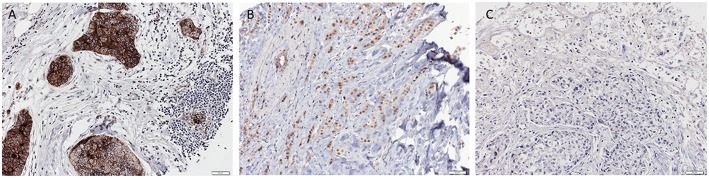
Representative pictures of triple negative breast cancer with strong **(A)**, moderate **(B)** and negative **(C)** Nectin-4 expression respectively (Magnification 200x).

For immunohistochemistry, 4 μm-sections were cut from the paraffin tissue block. Sections were pretreated with MW Tris/EDTA at 98°C for 30 min for antigen retrieval and then incubated with the primary Nectin-4 antibody (Abcam, ab 192033) overnight at 4°C. DAB was used as chromogen, and counterstaining was performed with Hematoxylin (Roche). Nectin-4 expression was detectable on the cell membrane. The Nectin-4 staining was scored according to the Quick score (QS) by using the following formula: QS = P (percentage of positive cells) × I (intensity), the maximum score being 300. The high Nectin-4 expression group was defined as a QS > 100 and the low Nectin-4 expression group as a QS = or < 100. The entire immunohistochemical analysis was performed by a trained research fellow blinded to the clinicopathological data, and challenging cases were reviewed and discussed together with an experienced breast pathologist, until consensus was reached. Representative pictures of high and low Nectin-4 expression can be found in Figure 1.

### Study Design

We performed a retrospective case selection of 148 primary human triple-negative breast cancers, which were available in our TMA platform. The clinical endpoint was overall survival, defined as percentage of patients in our study group who were alive after the follow-up period. The initially collected variables were median tumor size, mean age at diagnosis, tumor stage, lymph node involvement, tumor grade, histologic subtype, intrinsic subtype, Ki-67, time of death.

### Statistical Analysis Methods

We defined two subgroups of high (QS > 100) and low (QS = or < 100) Nectin-4 expression. To correlate Nectin-4 expression with clinicopathological features, we used Chi-Square or Mann-Whitney *U*-tests, respectively for categorical and non-categorical variables. Overall survival was calculated using the Kaplan-Meier method and differences between groups assessed using log-rank tests.Univariate analyses of the effect of high Nectin-4 expression and of high Nectin-4 expression stratified by intrinsic subtypes were performed using the Cox proportional hazards regression model. For multivariate Cox proportional hazards regression analyses, we evaluated the effects of clinicopathological parameters (age, tumor stage, lymph node involvement, tumor grade), intrinsic subtype and high Nectin-4 expression on overall survival. Hazard ratios and corresponding 95% confidence intervals were estimated. All tests were two-sided. *P*-values <0.05 were considered statistically significant. All analyses were performed using R v3.4.2. Any missing clinico-pathological information was assumed to be missing at random.

## Results

### Patient Characteristics

Table 1 shows basic demographic data. Mean age at diagnosis was 62 years (±15 years SD) and median tumor size was 25 mm (± 19 mm SD). The majority of the patients (53.4%) presented with a tumor stage pT2. Almost a third of the patients (29.1%) presented with a pT1 tumor stage. 51.4% of patients had no lymph node involvement, while 28.4% presented with a pN1 lymph node stage. Tumor grade 3 could be observed in the majority of the patients (78.4%). Invasive ductal carcinoma was the most observed histological subtype in 90% of the cases.

**Table 1:** Basic demographic data of all evaluable breast cancer cases (*n* = 148).

	**Number (*n*)**	**Percentage (%)**
Median tumor size (mm) ± SD	25 ± 19	
Mean age at diagnosis (years) ± SD	62 ± 15	
**Tumor stage**		
pT1	43	29.1
pT2	79	53.4
pT3	13	8.8
pT4	12	8.0
NA	1	0.7
**Lymph node involvement**		
pN0	76	51.3
pN1	42	28.4
pN2	14	9.5
pN3	10	6.7
NA	6	4.1
**Tumor grade**		
1	6	4.1
2	25	16.9
3	116	78.3
NA	1	0.7
**Histologic subtype**		
Invasive ductal	133	89.8
Invasive lobular	5	3.4
Mucinous	0	0.0
Apocrine	0	0.0
Cribriform	0	0.0
Others	10	6.8
NA	0	0.0

### Association Between Nectin-4 and Clinicopathological Parameters

High Nectin-4 expression (Nectin-4 high group) was found in in 86 (58%) of 148 cases. The association between high Nectin-4 expression and clinicopathological parameters is shown in Table 2. Mean age and tumor grade did not differ significantly between the Nectin-4-high and Nectin-4-low group, while the distribution of tumor stage was significantlly different between the two groups (*p* = 0.025), with Nectin-4-high being associated with a lower tumor stage. Furthermore, lymph node involvement was significantlly different between the two groups (*p* = 0.034), with high Nectin-4 expression being more frequent in patients with pN0 lymph node stage.

**Table 2:** Association between Nectin-4 expression and clinicopathological parameters.

**Clinicopathologic parameter**	**Nectin-4 high**		**Nectin-4 low**		***p*-value**
Mean tumor size (mm) ± SD	25 ± 20		30 ± 20		0.125
Mean age at diagnosis (years) ± SD	64 ± 14		60.5 ± 15		0.765
	**(*n*)**	**(%)**	**(*n*)**	**(%)**	
Tumor stage					0.025
pT1	28	32.9	1	3.8	
pT2	43	50.6	18	69.2	
pT3	8	9.4	3	11.5	
pT4	6	7.1	4	15.5	
Lymph node involvement					0.034
pN0	42	64.0	14	49.5	
pN1	28	31.3	4	39.4	
pN2	5	4.7	6	11.1	
pN3	7	0.0	1	0.0	
Tumor grade					0.722
1	2	2.3	0	0.0	
2	15	17.4	5	20.0	
3	69	80.3	20	80.0	

### Prognostic Significance

**Figure 2:**
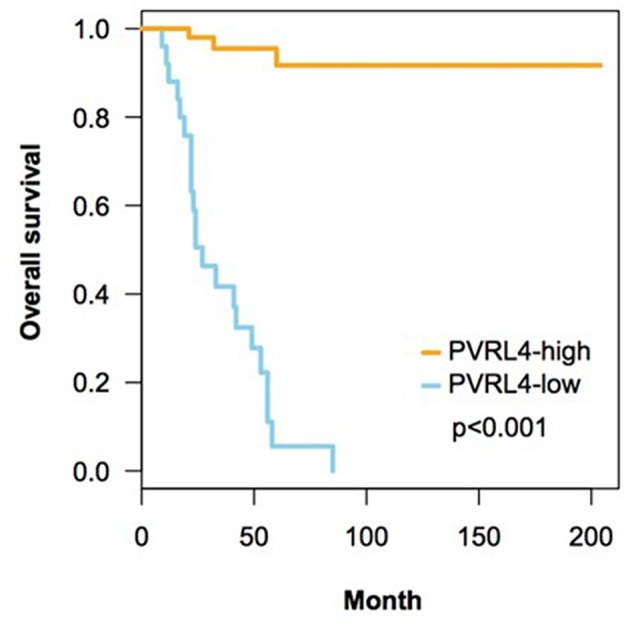
Correlation of expression of Nectin-4 on overall survival. This figure contains Kaplan-Meyer plots depicting the impact of high Nectin-4 expression on overall survival. Statistical analyses were performed using log-rank tests.

In univariate survival analysis, high Nectin-4 expression was associated with a significantly better overall survival (Hazard ratio 0.271, 95% CI 0.0077–0.0952, *p* < 0.001, Table 3). In multivariate analyses for the effect of clinicopathological parameters and high expression of Nectin-4 on overall survival, age (Hazard ratio 1.0369, 95% CI 1.0068–1.0678, *p* = 0.0158) and high Nectin-4 expression (Hazard ratio 0.0220, 95% CI 0.0055–0.0889, *p*<0.001) were associated with significantly better overall survival (Table 4 and Figure 2).

**Table 3:** Univariate analyses for the effect of Nectin-4 expression on overall survival.

**Nectin-4 expression, all cases**	**Hazard Ratio (95% CI)**	***p*-value**
Nectin-4 high	0.0271 (0.0077–0.0952)	<0.001

**Table 4:** Multivariate analysis for the effect of clinicopathologic parameters and Nectin-4 expression on overall survival.

**Clinicopathologic parameter**	**Hazard ratio (95% CI)**	***p*-value**
Age (per 1-year)	1.0369 (1.0068–1.0678)	0.0158
Tumor stage	1.4357 (0.9228–2.2338)	0.1088
Lymph node involvement	1.6303 (0.9975–2.6644)	0.0512
**Nectin-4 expression**		
Nectin-4 high	0.0220 (0.0055–0.0889)	<0.0001

## Discussion

Our results show that Nectin-4 expression is an independent prognostic biomarker for better overall survival in TNBC. To our knowledge, this is the first study with a sample size as large as this to investigate Nectin-4 expression in human TNBC. Interestingly, high Nectin-4 expression showed a significant association with lower tumor stage and negative lymph nodes.

Several reports identified Nectin-4 as a new promising tumor antigen in various carcinomas (14–19). Most recently, M-Rabet et al. performed a mRNA- and protein-based analysis of Nectin-4 expression in approximately 5,700 invasive breast cancer samples, showing that Nectin-4 is significantly overexpressed in both triple negative and basal breast cancer samples, with high expression of mRNA being an independent biomarker associated with poor prognosis in TNBC (21). Within the same cohort, protein expression of Nectin-4 was analyzed by immunohistochemistry in 61 TNBC cases and positively correlated with mRNA expression. Two other studies investigated Nectin-4 expression by immunohistochemistry in luminal-A breast cancers (22) and in a mixed cancer cohort (19). Lattanzio et al. (22) showed a significant association of high membranous expression of Nectin-4 with lower disease-free survival as well as lower distant-free survival in the luminal-A breast cancer cohort (T1 and T2, *n* = 193). Challita-Eid et al. (19) conducted immunohistochemical staining of Nectin-4 on 2394 patient specimens from different tumor entities including cancer of the bladder, breast, lung, pancreas, ovaries, head/neck and esophagus). A positive staining for Nectin−4 was detected in 69% of all specimen. When moderate Nectin-4 expression was defined as a QS > 100 and strong expression as a QS > 200, immunohistochemical analysis of 36 healthy human organs showed homogenous weak to moderate staning, including in the breast. Interestingly, moderate (26%) and strong (27%) Nectin-4 expression was seen most frequently in bladder cancer, followed by breast cancer (53%, *n* = 654). Whereas 30% of the invasive ductal carcinomas had strong Nectin-4 expression, only 20% of the invasive lobular carcinomas were categorized into this group. In 18% of cancer metastases, strong Nectin-4 expression could be observed. There was no specific investigation of TNBC or association of Nectin-4 expression with overall or recurrence free survival in this study.

In contradiction to the results of M-Rabet et al. (21), our results indicate that high Nectin-4 expression is associated with a better overall survival in TNBC. Our analysis is based on protein expression as determined by immunohistochemistry, whereas M-Rabet et al. analyzed mRNA expression by microarray technology. In their study high Nectin-4 expression in a breast cancer cohort of mixed molecular subtypes and also specifically in TNBC was associated with a lower metastasis free survival. Analyses per molecular subtype indicated a significant association only for TNBC. In contrast, our multivariate analysis shows that high Nectin-4 expression is significantly associated with better overall survival (hazard ratio 0.22 in TNBC). In both series, adjuvant treatment was not specified and comparison of the underlying cohorts is hampered by lack of full clinical data.

Due to the fact that Nectin-4 is mainly expressed during fetal development with a decrease of expression in adult tissues (23), its re-expression during tumor development makes it a tumor-associated antigen with the possibility of developing a targeted therapy. To our knowledge, no studies investigating Nectin-4 expression during progression of cancer exist. Association of Nectin-4 expression with markers of tumor proliferation was analyzed in pancreatic cancer patients (18). Additionally, a significant inhibition of cell proliferation in human pancreatic cells by siRNA-mediated gene silencing could be demonstrated *in vitro*.

Challita-Eid et al. (19) observed strong membranous Nectin-4 expression in only 18% of the investigated metastases, while it was more often observed in primary tumors. One possible explanation could be that Nectin-4 expression on the cancer cell surface is highly present during tumor formation, but declines during progression. This would explain the better association between high Nectin-4 expression and its association with lower tumor stage and absent lymph node involvement. Furthermore, expression on a DNA or mRNA level might not have the same impact and could still be measured in progressive disease, while the surface protein is not longer necessarily expressed in advanced stages.

While presenting important findings, our study also has several limitations. First, even though the cohort is well-characterized, it is based on a retrospective analysis. Secondly, we did not investigate the role of high expression of Nectin-4 in other molecular breast cancer subtypes and not on a DNA or mRNA level. In addition, our core diameter of 0.6 mm is small, and we did not use duplets of the cancers. However, several studies have shown a high concordance for immunohistochemical stainings between TMA and whole slides sections, even for a core diameter of 0.6 mm (24–28). Finally, the exact molecular mechanisms which could potentially improve prognosis in Nectin-4 expressing TNBC are not established. Further translational studies are needed to investigate the role of membranous Nectin-4 expression during cancer progression and to reveal potential interactions with the immune system or therapeutic interventions.

Despite conflicting results, our data add insightful information to the prognostic significance of high Nectin-4 expression in TNBC. Nectin-4 represents a potential target in TNBC, and its role in molecularly defined breast cancer subtypes should be investigated in larger patient cohorts.

## Disclosure

All authors state that they have no financial competing interests in relation to this manuscript.

## Data Availability

The datasets generated for this study are available on request to the corresponding author.

## Ethics Statement

The studies involving human participants were reviewed and approved by Ethikkommission Nordwest- und Zentralschweiz (EKNZ). Written informed consent for participation was not required for this study in accordance with the national legislation and the institutional requirements.

## Author Contributions

SS and SM planned and designed the study, and interpreted the data. JZ and SM analyzed the immunohistochemical stainings and both wrote part of the manuscript. SP and CN performed the data analysis and created Figure 2. SM created Figure 1. SS created the tables. WW, CK, AI, and RM critically revised the article for important intellectual content. All authors approved of the version to be published and agree to be accountable for all aspects of the work in ensuring that questions related to the accuracy or integrity of the work are appropriately investigated and resolved.

### Conflict of Interest Statement

The authors declare that the research was conducted in the absence of any commercial or financial relationships that could be construed as a potential conflict of interest.
